# Virome assembly reveals draft genomes of native Pseudomonas phages isolated from a paediatric bronchoalveolar lavage sample

**DOI:** 10.1128/mra.01030-24

**Published:** 2024-12-20

**Authors:** Patricia Agudelo-Romero, Jose A. Caparros-Martin, Abhinav Sharma, Montserrat Saladié, Peter D. Sly, Stephen M. Stick, Fergal O'Gara, COMBAT study group

**Affiliations:** 1 Wal-Yan Respiratory Research Centre, Telethon Kids Institute, Perth, Western Australia, Australia; 2 Australian Research Council Centre of Excellence in Plant Energy Biology, School of Molecular Sciences, The University of Western Australia, Perth, Western Australia, Australia; 3 European Virus Bioinformatics Center, Friedrich-Schiller-Universitat Jena, Thuringia, Germany; 4 Curtin Health Innovation Research Institute (CHIRI), Curtin University, Perth, Western Australia, Australia; 5 UWA Medical School, The University of Western Australia, Perth, Western Australia, Australia; 6 DSI-NRF Centre of Excellence for Biomedical Tuberculosis Research, SAMRC Centre for Tuberculosis Research, Division of Molecular Biology and Human Genetics, Faculty of Medicine and Health Sciences, Stellenbosch University, Cape Town, South Africa; 7 Children’s Health and Environment Program, Child Health Research Centre, The University of Queensland, Brisbane, Australia; 8 Department of Respiratory and Sleep Medicine, Perth Children’s Hospital, Perth, Western Australia, Australia; 9 Centre for Cell Therapy and Regenerative Medicine, School of Medicine and Pharmacology, The University of Western Australia and Harry Perkins Institute of Medical Research, Perth, Western Australia, Australia; 10 BIOMERIT Research Centre, School of Microbiology, University College Cork, Cork, Ireland; Portland State University, Portland, Oregon, USA

**Keywords:** viruses, cystic fibrosis, bronchoalveolar lavage, metagenomics

## Abstract

We present lung virome data recovered through shotgun metagenomics in bronchoalveolar lavage fluid from an infant with cystic fibrosis, who tested positive for *Stenotrophomonas maltophilia* infection. Using a bioinformatic pipeline for virus characterization in shotgun metagenomic data, we identified five viral contigs representing Pseudomonas phages classified as Caudoviricetes.

## ANNOUNCEMENT

Cystic fibrosis (CF) is a genetic condition that disrupts airway physiology, making patients susceptible to lung infections and chronic inflammation (1). While CF research has characterized lung bacterial communities, the associated virome remains understudied. Characterizing the lung virome is essential for understanding microbiome dynamics and its impact on respiratory health in CF. We present five viral contigs (vContigs) from shotgun metagenomic data in bronchoalveolar lavage fluid (BALF) of a CF infant with a *Stenotrophomonas maltophilia* lung infection (2).

This study represents an exploratory outcome of the COMBAT CF clinical trial (Clinicaltrials.gov: NCT01270074) (3). The COMBAT CF study protocol was approved by site-specific hospital Human Research Ethics Committees (HREC) and the HREC at the University of Western Australia reviewed this study and granted ethics exemption (2024/E000843). For nucleic acid extraction, 2 mL of BALF was centrifuged at 20,000 × *g* for 30 min at 4°C. Pellets underwent enzymatic digestion (MetaPolyzyme and proteinase K), followed by bead-beating, and chloroform:isoamyl alcohol extraction. Nucleic acids were then precipitated from the aqueous phase with polyethylene glycol, centrifuged using the same conditions as above, washed with ethanol, and resuspended in sterile water (2, 4). Libraries were built using the Nextera XT kit (Illumina, San Diego, CA, USA), and sequenced using a 150 bp pair-end configuration in a NovaSeq 6000 (Illumina) instrument at Genewiz (China). We retained 154 million reads with a mean Phred-like *Q*-score greater than or equal to 35 (5). We used the Snakemake pipeline EVEREST-meta for virus discovery (https://github.com/agudeloromero/EVEREST_meta)(6), using the database v4 (November 2024) (7), as we described (8, 9). All tools were run with default parameters unless otherwise specified.

Human reads were removed with minimap2 v2.24 (10), followed by deduplication and digital normalization using BBMAP v38.96 (11). We retained 2,559,801 non-human reads, which were *de novo* assembled with SPAdes v3.13.0 (12). Subsequently, viral contigs longer than 5,000 bp were retained using VirSorter v2.2.3 (13). Quality genome assessment was performed using CheckV v0.9.0 (14). EVEREST-meta uses the Lowest Common Ancestor algorithm from MMSeq2 v13.45111 taxonomy tool (15, 16), using NCBI (nucleotide; nt) and UniProt (amino acid; aa) databases for viral taxonomic classification and to obtain the closest related virus. Complementary analyses were performed for functional annotation with Pharokka v1.3.0 (17), virulence and antibiotic resistance gene identification using ABRICATE v1.0.1 (https://github.com/tseemann/abricate) (18) with the VFDB (July 2019) (19) and CARD (July 2019) (20) databases, average nucleotide identity by orthology (OrthoANI) v0.6.0 (21), and host prediction using iPHoP v1.2.0 (22).

Five vContigs were characterized with a length between 25,966 and 5,318 bp and 60.11% average GC content (Table 1). Notably, no vContigs contained virulence or antimicrobial resistance genes, while the predicted genes were primarily related to DNA, RNA, nucleotide metabolism, head, and packaging biological processes, as well as unknown function (Table 1). The five vContigs were classified as Caudoviricetes using both NCBI and UniProt databases. Using UniProt, the closest related phage for all vContigs were *Pseudomonas* phages. Host prediction supported *Pseudomonas* as the potential host (Table 1). No phages associated with *S. maltophilia* were detected.

**TABLE 1:** Five viral contigs (vContigs) were recovered from shotgun metagenomic data from DNA from BALF of an infant with cystic fibrosis^*a*^

Contigs	vContig_1	vContig_2	vContig_3	vContig_4	vContig_5
Genome size (bp)	25,966	14,256	12,689	7,952	5,318
Genome coverage (×)	34.1506	13.8512	38.1961	9.2591	12.422
No. of mapped reads	5,816	1,291	3,193	493	440
GC content (%)	59.43	60.96	60.71	60.48	58.99
CheckV completeness (%)	57.36	27.71	24.79	16.93	11.63
CDS*	39	22	30	10	11
Connector*	3	2	0	0	0
DNA, RNA, and nucleotide metabolism*	2	0	6	1	2
Head and packaging*	6	3	0	0	0
Integration and excision*	1	0	1	0	0
Lysis*	2	1	0	0	0
Moron, auxiliary metabolic gene, and host takeover*	0	0	1	0	0
Other*	0	1	1	0	1
Tail*	8	2	0	6	0
Transcription regulation*	0	1	1	0	1
Unknown function*	17	12	20	3	7
tRNAs, CRISPRs, tmRNAs*	0	0	0	0	0
Virulence factors (VFDB)	0	0	0	0	0
AMR genes (CARD)	0	0	0	0	0
Lowest common ancestor by nucleotide (NCBI) database	Class: Caudoviricetes	Class: Caudoviricetes	Class: Caudoviricetes	Class: Caudoviricetes	Class: Caudoviricetes
Closest-related phage NBCI, nt (GenBank accession #)	Pseudomonas phage PS-1 (NC_029066.1)	Pseudomonas phage PS-1 (NC_029066.1)	Pseudomonas virus D3 (NC_002484.2)	Ralstonia phage Dina (NC_055026.1)	Pseudomonas phage JBD44 (NC_030929.1)
Average nucleotide identity by orthology (OrthoANI) to the closest-related phage	75.74	87.80	71.62	61.36	79.21
Lowest common ancestor by amino acid (UniProt; TrEMBL) database	Class: Caudoviricetes	Class: Caudoviricetes	Class: Caudoviricetes	Class: Caudoviricetes	Class: Caudoviricetes
Closest-related phage UniProt, aa (UniProtKB accession #)	Pseudomonas phage PS-1 (A0A0H5ART3)	Pseudomonas phage PS-1 (A0A0H5AWC7)	Pseudomonas phage vB_PeaS_FBPa47 (A0A9E7QP96)	Pseudomonas phage PMBT14 (A0A2S1B6B3)	Pseudomonas phage JBD44 (A0A125RNK2)
Host prediction, genus level	Pseudomonas	Pseudomonas	Pseudomonas	Pseudomonas	Pseudomonas
Confidence score of host prediction	94	94.9	96.9	93.9	96.3
GenBank accession number	PP986815	PP986816	PP986817	PP986818	PP986819

^*a*^ Functional annotations performed with Pharokka (17), are indicated with an asterisk.

## Data Availability

This Whole Genome Shotgun project has been deposited in the BioProject PRJNA1126024; GenBank accession numbers PP986815, PP986816, PP986817, PP986818, and PP986819; BioSample SAMN41940487; Sequence read archive accession number SRR29521294.
